# Supplementary material for: Composite GO/Ceramic Membranes Prepared via Chemical Attachment: Characterisation and Gas Permeance Properties
Source: Membranes (Basel). 2022 Nov 24;12(12):1181. doi: 10.3390/membranes12121181 (PMC9787500; doi:10.3390/membranes12121181)
Supplement: Supplementary file 1 [file membranes-12-01181-s001.zip › membranes-2035737-supplementary.pdf]

Table S1: Pressure (mbar)-permeance (mol/m²/s/Pa) of He for T=25°C, 60°C and 100°C.

	He	He	He	He	He	He	He	He	He	He	He	He	He	He	He
**T(°C)**	**25**	**25**	**25**	**25**	**25**	**60**	**60**	**60**	**60**	**60**	**100**	**100**	**100**	**100**	**100**
**Al_2O_3 blank P(mbar)**	44.375	91.25	137.5	183.125	228.125	45	92.5	141.875	190	236.25	49.375	99.375	150.625	200	250
**permeance (mol/m²/s/Pa)**	3.63E-06	3.67E-06	3.70E-06	3.70E-06	3.68E-06	3.56E-06	3.55E-06	3.55E-06	3.57E-06	3.59E-06	3.24E-06	3.30E-06	3.33E-06	3.38E-06	3.41E-06
**Al_2O_3 APTES GO**	100	149,375	198.125	246.875	295	113,125	169.375	225	281.25	336.875	124.375	189.375	251.875	315	376.875
	1.70E-06	1.72E-06	1.74E-06	1.74E-06	1.75E-06	1.46E-06	1.49E-06	1.51E-06	1,52E-06	1.53E-06	1.33E-06	1.32E-06	1.36E-06	1.36E-06	1.379E-06
**Al_2O_3 APTES GO-F**	41.875	72.5	145.625	173.125	214.375	104.375	173.75	355	406.25	489.375	142.5	247.5	400.625	476.25	567.5
	1.11E-06	1,13E-06	1.20E-06	1.22E-06	1.24E-06	4.69E-07	4.74E-07	4.96E-07	4.99E-07	5.17E-07	2.93E-07	3.12E-07	3.31E-07	3.53E-07	3.57E-07
**Al_2O_3 GLYMO GO**	170	340	400	495	600	250	510	610	770	1000	244.375	337.5	423.75	516,875	601.875
	4.63E-07	4.87E-07	5.01E-07	5.07E-07	5.65E-07	3.18E-07	3.25E-07	3.28E-07	3.26E-07	3.39E-07	1.72E-07	1.72E-07	1.84E-07	1.85E-07	1.88E-07
**Al_2O_3 GLYMO GO-F**	70.625	143.125	210.625	279.375	346,25	150	305.625	456.875	601.875	742.5	236.875	476.875	710.625	933.75	1150
	1.19E-06	1.20E-06	1.20E-06	1.23E-06	1.23E-06	5.69E-07	5.72E-07	5.78E-07	5.85E-07	5.95E-07	3.66E-07	3.76E-07	3.77E-07	3.81E-07	3.84E-07
**Al_2O_3 PDA GO**	68.125	136.25	202.5	268	324.375	87.5	169.375	250.625	329.375	406.875	97.5	195.625	291.875	387.5	481.25
	2.54E-06	2.56E-06	2.59E-06	2.62E-06	2.72E-06	1.99E-06	2.05E-06	2.09E-06	2.14E-06	2.16E-06	1.78E-06	1.78E-06	1.79E-06	1.81E-06	1.82E-06
**Al_2O_3 PDA rGOT**	50.625	101.875	151.875	202.5	251.875	55.625	111.25	165	218.125	274.375	58.75	118.125	178.125	236.25	294.375
	3.41E-06	3.411E-06	3.42E-06	3.44E-06	3.48E-06	3.10E-06	3.12E-06	3.18E-06	3.19E-06	3.20E-06	2.92E-06	2.94E-06	2.95E-06	2.95E-06	2.96E-06
**ZrO_2 blank**	105.38	203.56	296.925	398.13	495.945	112.65	204.438	305.12	396.112	507.264	106.03	197.372	299.978	397.816	529.387
	1.09E-06	1.07E-06	1.31E-06	1.47E-06	1.60E-06	8.26E-07	9.48E-07	1.16E-06	1.15E-06	1.39E-06	6.94E-07	8.24E-07	9.42E-07	1.03E-06	1.18E-06
**ZrO_2 APTES GO**	109.79	207.572	319.567	393	536.495	113.761	202.72	299.951	401.328	528.487	106.05	200.541	303.61	401.521	498.78
	1.49E-07	1.52E-07	1.52E-07	1.54E-07	1.56E-07	1.31E-07	1.31E-07	1.33E-07	1.35E-07	1.37E-07	1.22E-07	1.21E-07	1.20E-07	1.19E-07	1.19E-07
**ZrO_2 GLYMO GO**	104.796	202.122	294.762	395.756	491.83	110.885	201.407	302.143	392.685	501.181	104.146	194.677	295.682	392.731	520.070
	2.47E-07	2.59E-07	2.69E-07	2.78E-07	2.91E-07	2.05E-07	2.12E-07	2.23E-07	2.29E-07	2.32E-07	1.43E-07	1.20E-07	1.17E-07	1.17E-07	1.72E-07
**ZrO_2 GLYMO GO-F**	110.25	216.392	310.645	412.711	517.852	115.77	218.31	314.96	420.51	525.70	121.788	218.86	317.39	408.58	540
	1.6E-08	1.69E-08	1.76E-08	1.80E-08	1.85E-08	2.72E-09	2.81E-09	2.87E-09	3.03E-09	3.13E-09	1.94E-09	2.01E-09	2.05E-09	2.31E-09	2.25E-09
**ZrO_2 PDA rGOT**	119.54	213.442	313.145	400.57	522.53	115.41	212.128	300.1769	413.547	514.44	115	214.168	318.6	411.4	500.7
	4.02E-08	4.23E-08	4.53E-08	4.78E-08	5.09E-08	2.46E-08	2.55E-08	2.60E-08	2.69E-08	2.63E-08	1.79E-08	1.85E-08	1.93E-08	1.98E-08	2.01E-08
**ZrO_2 PDA GO**	127.804	217.547	313.641	416.199	522.012	130.018	225.200	318.872	419.826	500.989	115.603	215.336	317.399	412.806	498.544
	4.89E-09	4.95E-09	5.02E-09	5.10E-09	5.21E-09	4.73E-09	4.74E-09	4.77E-09	4.92E-09	4.97E-09	1.88E-09	1.91E-09	1.98E-09	2.01E-09	2.05E-09
**Al_2O_3 PDA**	55	109.375	163.125	215.625	267.5	60	119.375	178.125	236.25	293.75	63.75	126.875	190	252.5	314.375
	2.96E-06	3.08E-06	3.13E-06	3.135E-06	3.17E-06	2.65E-06	2.75E-06	2.78E-06	2.82E-06	2.83E-06	2.46E-06	2.55E-06	2.6E-06	2.62E-06	2.609E-06

Table S2: Pressure (mbar)-permeance (mol/m²/s/Pa) of CO_2 for T=25°C, 60°C and 100°C.

	CO_2	CO_2	CO_2	CO_2	CO_2	CO_2	CO_2	CO_2	CO_2	CO_2	CO_2	CO_2	CO_2	CO_2	CO_2
**T(°C)**	**25**	**25**	**25**	**25**	**25**	**60**	**60**	**60**	**60**	**60**	**100**	**100**	**100**	**100**	**100**
Al_2O_3 blank **P(mbar)**	66.875	133.75	205	266.875	329.375	74.375	151.875	226.25	295	364.375	85.625	165.625	246.25	322.5	396.25
**permeance (mol/m²/s/Pa)**	1.194E-06	1.18E-06	1.13E-06	1.207E-06	1.225E-06	1.057E-06	1.049E-06	1.04E-06	1.1067E-06	1.09E-06	9.60E-07	9.246E-07	9.33E-07	9.85E-07	1.01E-06
Al_2O_3 APTES GO	138.125	203.75	267.5	328.75	390.625	163.125	239.375	314.375	389.375	461.25	188.125	275	362.5	447.5	530
	6.04E-07	6.2E-07	6.41E-07	6.487E-07	6.59E-07	4.7E-07	5.12E-07	5.3E-07	5.21E-07	5.54E-07	3.984E-07	4.32E-07	4.65E-07	4.69E-07	4.78E-07
Al_2O_3 APTES GO-F	81.875	133.125	163.125	193.75	235	112.5	175.625	298.75	367.5	435.625	189.375	309.375	478.75	585	692.5
	4.27E-07	4.71E-07	4.95E-07	5.121E-07	5.30E-07	2.023E-07	2.030E-07	2.056E-07	2.1508E-07	2.228E-07	1.134E-07	1.131E-07	1.278E-07	1.27E-07	1.39E-07
Al_2O_3 GLYMO GO	180	430	500	620	800	400	700	830	1000	1200	600	1100	1270	1600	1980
	1.89E-07	1.91E-07	1.92E-07	1.98E-07	2.07E-07	8.86E-08	1.16E-07	1.19E-07	1.24E-07	1.39E-07	6.62E-08	7.47E-08	7.61E-08	7.799E-08	8.08E-08
Al_2O_3 GLYMO GO-F	88.125	168.125	244.375	320.625	394.375	165	315	455.625	592.5	722.5	271.25	429.375	520	606.875	742.5
	4.192E-07	4.905E-07	5.18E-07	5.33E-07	5.44E-07	2.2E-07	2.59E-07	2.75E-07	2.88E-07	2.97E-07	1.39E-07	1.59E-07	1.60E-07	1.65E-07	1.72E-07
Al_2O_3 PDA GO	51.875	109.375	175	243.75	315	71.875	148.125	231.875	318.75	408.75	104.375	208.75	315	423.75	533.75
	1.59E-06	1.56E-06	1.48E-06	1.41E-06	1.38E-06	1.15E-06	1.16E-06	1.12E-06	1.08E-06	1.06E-06	7.902E-07	8.28E-07	8.25E-07	8.21E-07	8.16E-07
Al_2O_3 PDA rGOT	75	146.25	216.25	282.5	348.125	80.625	158.75	235.625	310	384.375	89.375	175	258.125	341.875	422.5
	1.10E-06	1.19E-06	1.22E-06	1.22E-06	1.24E-06	1.01E-06	1.071E-06	1.09E-06	1.11E-06	1.134E-06	9.22E-07	9.72E-07	9.98E-07	1.01E-06	1.03E-06
ZrO_2 blank	103.572	153.177	206.92	250.405	302.11	110.12	207.251	307.731	408.994	517.07	50	100	120	150	180
	4.50E-07	5.18E-07	5.94E-07	6.34E-07	6.56E-07	3.94E-07	5.17E-07	5.91E-07	6.64E-07	7.45E-07	2.59E-07	3.18E-07	3.33E-07	3.79E-07	3.83E-07
ZrO_2 APTES GO	115.462	214.624	313.139	415.937	522.802	116.578	215.103	316	416.116	520.342	104.531	208.804	306	406.696	505.776
	4.43E-08	4.65E-08	4.72E-08	4.89E-08	4.99E-08	4.42E-08	4.30E-08	4.34E-08	4.39E-08	4.54E-08	3.75E-08	3.76E-08	3.79E-08	3.85E-08	3.88E-08
ZrO_2 GLYMO GO	103.13	152.319	205.503	248.409	300.072	108.601	204.962	304.848	405.962	512.688	50	100	120	150	180
	8.30E-08	8.73E-08	8.98E-08	9.23E-08	9.52E-08	6.86E-08	7.57E-08	7.95E-08	8.30E-08	8.59E-08	4.25E-08	3.85E-08	3.78E-08	3.64E-08	4.41E-08
ZrO_2 GLYMO GO-F	112.26	210.75	314.6	412.2	508.5	127.03	223.36	325.9	420.68	505.12	126.51	227.77	327.656	419.68	507.27
	5.41E-09	6.25E-09	7.19E-09	7.97E-09	8.72E-09	1.39E-09	1.04E-09	1.17E-09	1.30E-09	1.39E-09	6.72E-10	7.39E-10	8.71E-10	9.11E-10	9.78E-10
ZrO_2 PDA	122.50	220.31	326.26	423.44	523.12	123.45	222.07	325.80	424.06	521.43	114.33	216.01	320.65	413.94	508.91

rGOT															
	1.49E-08	1.82E-08	2.21E-08	2.57E-08	2.88E-08	9.42E-09	1.09E-08	1.26E-08	1.37E-08	1.51E-08	5.83E-09	6.32E-09	6.88E-09	7.18E-09	7.69E-09
ZrO_2 PDA GO	124.292	219.546	316.664	422.756	526.813	118.125	219.099	319	414.840	522.778	113.643	217.019	321.922	415.297	509.395
	1.47E-09	1.53E-09	1.58E-09	1.66E-09	1.70E-09	1.29E-09	1.34E-09	1.37E-09	1.42E-09	1.44E-09	6.03E-10	6.58E-10	7.17E-10	7.69E-10	8.60E-10
Al_2O_3 PDA	77.5	148.75	220	289.375	357.5	88.125	170	249.375	328.125	405	95.625	185.625	274.375	361.25	446.875
	8.3E-07	1.09E-06	1.135E-06	1.13E-06	1.17E-06	8.04E-07	9.56E-07	9.90E-07	1.01E-06	1.02E-06	7.05E-07	8.35E-07	8.61E-07	9.03E-07	9.18E-07

Table S3: Pressure (mbar)-permeance of (mol/m²/s/Pa) CH_4 for T=25°C, 60°C and 100°C.

	CH_4	CH_4	CH_4	CH_4	CH_4	CH_4	CH_4	CH_4	CH_4	CH_4	CH_4	CH_4	CH_4	CH_4	CH_4
**T(°C)**	**25**	**25**	**25**	**25**	**25**	**60**	**60**	**60**	**60**	**60**	**100**	**100**	**100**	**100**	**100**
Al_2O_3 blank **P(mbar)**	46.25	91.25	136.25	180	223.75	51.25	99.375	148.125	196.875	242.5	56.25	108.125	161.25	212.5	263.125
**permeance (mol/m²/s/Pa)**	1.92E-06	1.97E-06	1.98E-06	2.04E-06	2.06E-06	1.701E-06	1.77E-06	1.84E-06	1.86E-06	1.89E-06	1.56E-06	1.62E-06	1,69E-06	1.71E-06	1.73E-06
Al_2O_3 APTES GO	98.125	141.87	185.62	229.375	273.125	114.375	166.25	218.125	270	320	130	188.75	248,75	306.875	365
	9.56E-07	1.006E-06	1.03E-06	1.03E-06	1.045E-06	8.26E-07	8.5E-07	8.58E-07	8.75E-07	8.92E-07	7.40E-07	7.68E-07	7,65E-07	7.69E-07	7.82E-07
Al_2O_3 APTES GO-F	42.5	63.125	120	140.625	174.375	95.625	143.125	267.5	315.625	385.625	143.75	225	425	503.75	618.75
	7.32E-07	7.82E-07	8.23E-07	8.29E-07	8.36E-07	3.167E-07	3.451E-07	3.629E-07	3.69E-07	3.731E-07	2.09E-07	2.176E-07	2,27E-07	2.289E-07	2.35E-07
Al_2O_3 GLYMO GO	140	280	340	420	550	250	500	600	700	900	286.875	369.375	438,125	512.5	582.5
	3.25E-07	3.3E-07	3.30E-07	3.33E-07	3.38E-07	1.83E-07	1.86E-07	1.87E-07	2.00E-07	2.09E-07	9.43E-08	9.30E-08	1,034E-07	1.09E-07	1.11E-07
Al_2O_3 GLYMO GO-F	65	120.625	176.87	228.75	281.25	129.375	240	343.125	436.875	533.75	203.125	378.75	586,25	751.875	928.125
	7.89E-07	8.178E-07	8.29E-07	8.46E-07	8.55E-07	3.93E-07	4.14E-07	4.26E-07	4.47E-07	4.507E-07	2.53E-07	2.55E-07	2,56E-07	2.60E-07	2.59E-07
Al_2O_3 PDA GO	38.125	76.875	118.125	161.25	206.875	56.875	106.875	161.25	216.25	273.75	74.375	145	216,25	291.25	363.75
	2.61-06	2.53E-06	2.4E-06	2.3E-06	2.31E-06	1.75E-06	1.82E-06	1.79E-06	1.77E-06	1.76E-06	1.32E-06	1.34E-06	1,35E-06	1.33E-06	1.32E-06
Al2O3 PDA rGOT	51.875	100	146.875	193.125	240	56.875	108.75	160.625	211.875	263.125	61.25	118.75	176,25	231.875	288.125
	1.92E-06	1.95E-06	1.977E-06	1.979E-06	1.99E-06	1.74E-06	1.79E-06	1.80E-06	1.81E-06	1.82E-06	1.62E-06	1.63E-06	1.64E-06	1.65E-06	1.67E-06
ZrO_2 blank	101.655	201.715	302.145	402.255	503.665	105.35	201.496	302.059	415.292	503.508	106.359	203.783	303.46	410.924	502.03
	6.68E-07	8.94E-07	1.03E-06	1.22E-06	1.25E-06	5.77E-07	7.70E-07	9.40E-07	1.06E-06	1.16E-06	5.27E-07	6.57E-07	7.51E-07	9.31E-07	1.01E-06
ZrO_2 APTES GO	113.754	213.679	322.65	421.533	524.549	109.026	211.365	308.425	410.576	512.699	107.018	208.145	310.085	415.218	516.325
	7.57E-08	7.72E-08	7.72E-08	7.91E-08	8.08E-08	7.45E-08	7.24E-08	7.09E-08	7.23E-08	7.31E-08	6.23E-08	6.16E-08	6.21E-08	6.33E-08	6.39E-08
ZrO_2 GLYMO GO	100.907	200.631	300.37	399.445	500.028	103.781	198.969	296.735	411.171	508.244	104.794	201.070	300.7	406.580	495.146
	1.36E-07	1.47E-07	1.55E-07	1.60E-07	1.66E-07	1.12E-07	1.20E-07	1.29E-07	1.34E-07	1.39E-07	7.86E-08	7.56E-08	7.48E-08	8.97E-08	1.18E-07
ZrO_2 GLYMO GO-F	113.4	215.7	319.3	417.82	515	120.91	215.05	314.368	419.47	532.83	126.04	223.25	326.47	423.742	534.3
	8.82E-09	1.00E-08	1.15E-08	1.26E-08	1.36E-08	1.47E-09	1.68E-09	1.87E-09	1.17E-09	2.22E-09	8.28E-10	9.45E-10	1.03E-09	1.10E-09	1.21E-09
ZrO_2 PDA rGOT	114.756	215.300	313.983	417.71	508.921	118.023	213.417	316.393	416.855	506.853	115.024	210.085	311.15	410.720	520.127
	2.32E-08	2.79E-08	3.27E-08	3.72E-08	4.14E-08	1.51E-08	1.71E-08	1.94E-08	2.12E-08	2.36E-08	9.76E-09	1.04E-08	1.13E-08	1.18E-08	1.24E-08
ZrO_2 PDA GO	121.086	214.169	314.050	418.536	504.773	122.502	225.296	322.365	423.556	525.284	115.777	209.016	312.297	409.318	521.571
	2.47E-09	2.56E-09	2.66E-09	2.73E-09	2.80E-09	2.41E-09	2.48E-09	2.54E-09	2.58E-09	2.69E-09	9.88E-10	1.05E-09	1.14E-09	1.21E-09	1.30E-09
Al_2O_3 PDA	63.125	120	176.25	231.875	286.25	66.875	128.125	188.75	248.75	307.5	70.625	136.875	202.5	266.25	330.625
	1.58E-06	1.66E-06	1.68E-06	1.7E-06	1.72E-06	1.5E-06	1.59E-06	1.59E-06	1.59E-06	1.56E-06	1.41E-06	1.46E-06	1.45E-06	1.47E-06	1.48E-06

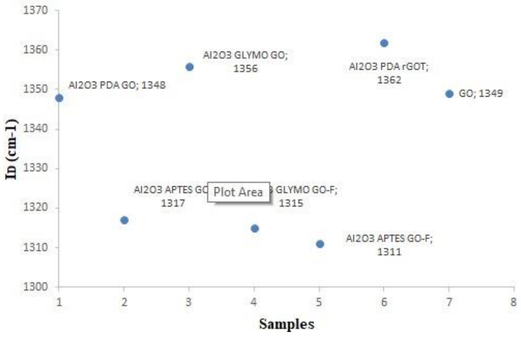

(a)

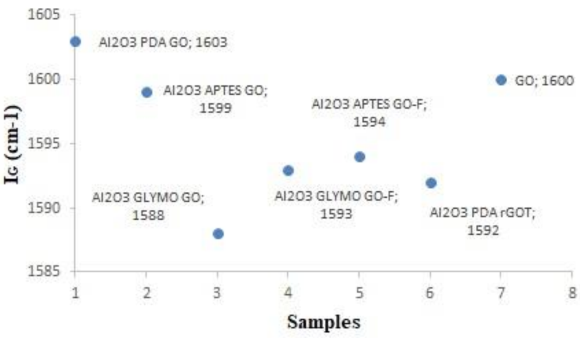

(b)

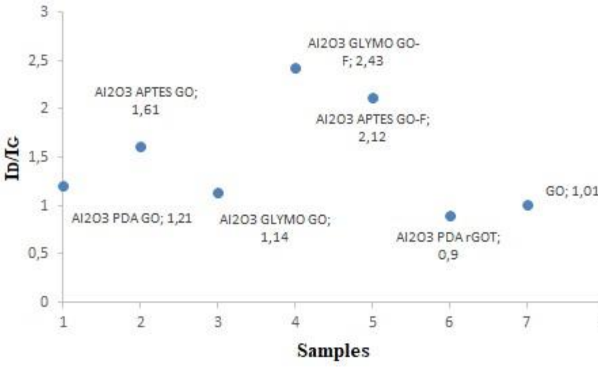

(c)

Figure S1: Intensities (a) I_D of D band and (b) I_G of G band and (c) integrated intensity ratio I_D/I_G of all membranes with different functionalization.

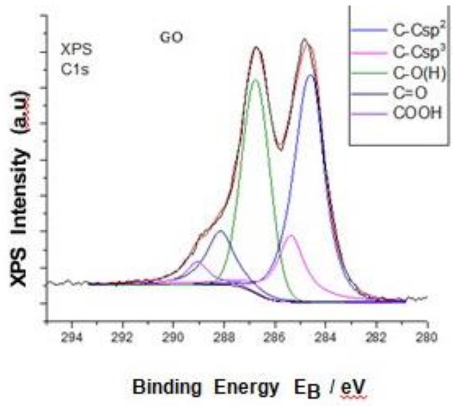

(a)

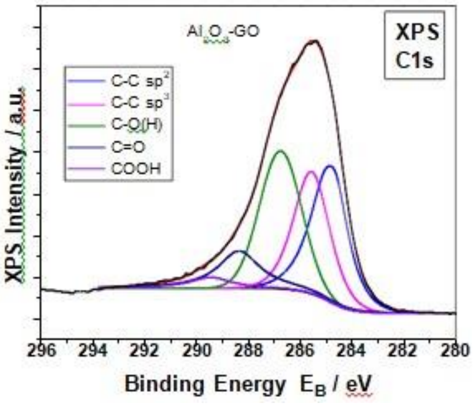

(b)

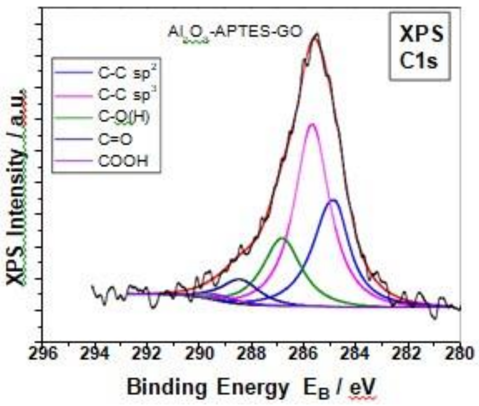

(c)

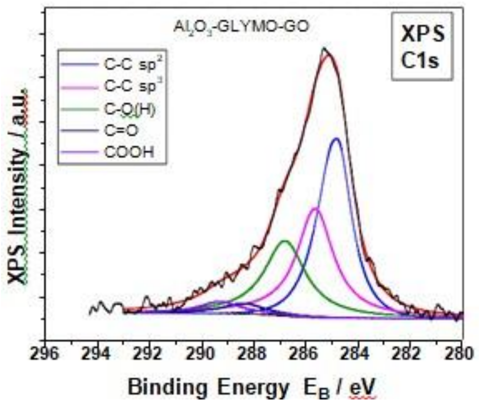

(d)

Figure S2: Deconvoluted C1s XPS spectra of (a) GO, (b) Al_2O_3 PDA GO, (c) Al_2O_3 APTES GO and (d) Al_2O_3 GLYMO GO.
